# Microbiological identification of pathogens in water from educational centers in Norte de Santander

**DOI:** 10.15649/cuidarte.4052

**Published:** 2025-04-30

**Authors:** Javier Andrés Soto, Karen Piedad Martinez-Marciales

**Affiliations:** 1 Universidad de Santander, Faculty of Medical and Health Sciences, Masira Research Institute, Cúcuta, Colombia. jav.soto@mail.udes.edu.co Universidad de Santander Universidad de Santander Cúcuta Colombia jav.soto@mail.udes.edu.co; 2 Universidad de Santander, Faculty of Medical and Health Sciences, Masira Research Institute, Cúcuta, Colombia. ka.martinez@mail.udes.edu.co Universidad de Santander Universidad de Santander Cúcuta Colombia ka.martinez@mail.udes.edu.co

**Keywords:** Coliforms, Food Safety, Water Monitoring, Coliformes, Inocuidad de los Alimento, Monitoreo del Agua, Coliformes, Inocuidade dos Alimentos, Monitoramento da Aguas

## Abstract

**Introduction:**

Water is an essential resource for survival, and therefore, its quality and safety must be a priority, especially for susceptible population groups.

**Objective:**

To determine the presence of mesophilic aerobes, coliforms, and Pseudomonas in drinking water in schools from three municipalities of Norte de Santander.

**Materials and Methods:**

Maintenance personnel were inquired about water storage. Samples from different sources were collected and processed using the membrane filtration method to identify aerobic mesophilic bacteria, E. coli, coliforms, Pseudomonas spp., and Salmonella spp. following the technical standards established for each microorganism.

**Results:**

Mesophilic bacteria growth was identified in 77.50% of the samples, total coliforms in 84.00%, fecal coliforms in 72.00%, and Escherichia coli in 21%. Pseudomonas spp. was also identified in 73.00% of the samples and Salmonella spp. in 10.50%.

**Discussion:**

These findings reflect non-compliance with current regulations due to the presence of indicator organisms such as mesophiles and the indicator par excellence in water quality: coliforms, a fact that is ratified by the presence of Pseudomonas spp. and Salmonella spp.

**Conclusion:**

The presence of these microorganisms is associated with failures in the water purification process, which allows us to expose the need for corrective actions to guarantee the microbiological quality of water and ensure health.

## Resumen

**Introducción:**

El agua es un recurso esencial para la supervivencia y por ello su calidad e inocuidad debe ser una prioridad, especialmente para grupos poblacionales susceptibles.

**Objetivo:**

Determinar la presencia de aerobios mesófilos, coliformes y Pseudomonas en agua de consumo en colegios de 3 municipios de Norte de Santander.

**Materiales y métodos:**

Se realizó indagación con el personal de mantenimiento sobre el almacenamiento del agua. Se recolectaron muestras de diferentes fuentes, las cuales se procesaron por el método de filtración por membrana para identificar bacterias Aerobias Mesófilas, E. coli, coliformes, Pseudomonas spp y Salmonella spp. mediante las normas técnicas establecidas para cada microorganismo.

**Resultados:**

Se identificó el crecimiento de bacterias Mesófilas en un 77,50%, coliformes totales y fecales en un 84,00% y 72,00% respectivamente y Escherichia coli en un 21%. También se identificó Pseudomonas spp. en un 73,00% y Salmonella spp. con 10,50%.

**Discusión:**

Estos hallazgos reflejan el incumplimiento de normativa vigente debido a la presencia de bacterias indicadoras como los mesófilos, y el indicador por excelencia en calidad de aguas: los coliformes; hecho que se ratifica con Pseudomonas spp. y Salmonella spp.

**Conclusiones:**

La presencia de estos microorganismos está asociada a fallas en el proceso de potabilización, permitiendo exponer la necesidad de acciones correctivas que permitan garantizar la calidad microbiológica del agua y la salud.

## Resumo

**Introdução:**

A água é um recurso essencial para a sobrevivência e, portanto, sua qualidade e segurança devem ser prioridade, especialmente para grupos populacionais suscetíveis.

**Objetivo:**

Determinar a presença de aeróbios mesófilos, coliformes e Pseudomonas na água potável em escolas de 3 municípios do Norte de Santander.

**Materiais e métodos:**

A equipe de manutenção foi questionada sobre o armazenamento de água. Amostras foram coletadas de diferentes fontes, as quais foram processadas pelo método de filtração por membrana para identificar bactérias aeróbicas mesófilas, E. coli, coliformes, Pseudomonas spp. e Salmonella spp. através das normas técnicas estabelecidas para cada microrganismo.

**Resultados:**

O crescimento de bactérias mesófilas foi identificado em 77,5%, coliformes totais e fecais em 84% e 72% respectivamente, e Escherichia coli em 21%. Pseudomonas spp. também foi identificada. em 73% e Salmonella spp. com 10,5%.

**Discussão:**

Estes achados refletem o não cumprimento das normas vigentes devido à presença de bactérias indicadoras como os mesófilos, e o indicador por excelência da qualidade da água: os coliformes; fato que é confirmado por Pseudomonas spp. e Salmonella spp.

**Conclusões:**

A presença desses microrganismos está associada a falhas no processo de purificação, permitindo expor a necessidade de ações corretivas para garantir a qualidade microbiológica da água e a saúde.

## Introduction

Water is an essential element for survival and is a need that directly influences health. Poor-quality water for human consumption is associated with diseases that arise from the lack of access to water sources and inadequate sanitation conditions. Schools located in rural areas often serve communities with a high prevalence of diseases related to inadequate water, sanitation, and hygiene conditions^1^. According to the United Nations Educational, Scientific and Cultural Organization (UNESCO), more than 800,000 people die each year from diarrhea caused by unsafe drinking water, poor sanitation, and inadequate hand hygiene, about 36% of whom are children under five years of age^2^. 

Drinking water is a basic service in any part of the world^3^. Ensuring its distribution and expanding access to potable water have been priorities monitored over the last few years^4^. It is necessary to validate water quality through in situ analysis of characteristics such as pH and chlorine levels, along with microbiological testing to identify any risk of contamination promptly^5^. 

In Mexico, in their study on the microbiological quality of drinking water in public schools, Iñiguez et al. found that 59% of the water samples analyzed exceeded permissible limits. While some samples met the required level of chlorine, others did not. Similarly, pathogens such as Escherichia coli were detected in 13.6% of the samples, and strains of Citrobacter freundii, Klebsiella spp., and Pseudomonas spp.^6^ were also isolated. In Venezuela, a study conducted in a context comparable to that of Iñiguez et al. identified contamination in nearly all the samples analyzed. The authors concluded that the indicator microorganisms found posed a health risk^7^. Continuing with evidence from Latin America, a study in Argentina on the microbiological quality of school drinking water revealed that 73% of the samples were unfit for consumption^8^.

These studies from Central and South America reveal coincidences regarding the lack of adequate water management and storage and the growing need for integrated water management in educational institutions. This is why research on water quality and safety is being conducted, particularly in Latin America, such as the research conducted by Mucinhato in 2022^9^. 

In Colombia, supplying potable water in rural areas is a challenge^10^. Enteric pathogens present in water pose a public health risk^11^,^12^. Global regulations infer the presence of these pathogens primarily through the analysis of fecal indicator organisms and recommend targeted studies of specific enteric genera only in high-risk areas. Water monitoring is critical, whether the source is a treated drinking water system or untreated water since water characteristics depend on external factors that exert selective pressure on the natural microbial population^13^, contributing to the proliferation of pathogenic microorganisms among vulnerable populations^14^. In our country, the Ministry of Health and Social Protection, the Ministry of Housing, City and Territory, the Superintendency of Household Public Utilities, and the National Health Institute publish the National Water Quality Report annually. This report serves as a reference for authorities and stakeholders involved in decision-making and implementing control measures^15^. 

The city of San José de Cúcuta reported a Water Quality Risk Index of 4.0 in 2020, placing it in the ‘no risk’ category^16^. However, the greatest microbiological risk associated with drinking water—and the primary focus of monitoring efforts—stems from a group of microorganisms known as coliforms. This group includes the genera Escherichia, Edwarsiella, Enterobacter, Klebsiella, Serratia, and Citrobacter. These microorganisms can be found in large quantities in surface waters, vegetation, and soils. Notably, spaces of Enterobacter and Klebsiella can colonize the inner surfaces of water pipes and storage tanks, where they form biofilms under conditions such as the presence of nutrients, warm temperatures, low disinfectant concentrations, and prolonged storage^17^. Biofilm formation in drinking water distribution systems is a widely studied topic^18^. Over time, bacterial communities inevitably establish themselves on the internal surfaces of pipelines, whether under conditions of high or low water flow or varying shear stress. Opportunistic pathogens can survive longer and exhibit greater resistance to chlorine when embedded within biofilms^3^. Coliform microorganisms and Escherichia coli are the best indicators of fecal contamination. Escherichia coli is traditionally used to assess the microbiological quality of water and continues to serve as a key parameter in monitoring and surveillance efforts^19^.

Pseudomonas spp., due to its high resistance to chlorine, is considered an indicator of water disinfection efficiency^20^. The presence of Salmonella spp. is particularly concerning because of its association with fecal contamination, high viability, and pathogenic potential over time^20^. In this context, the objective of the present study was to detect the presence of Escherichia coli, Salmonella spp., Pseudomonas spp., and mesophilic bacteria in drinking water from 20 schools located in three municipalities of Norte de Santander, with the aim of learning about the quality of the water in these key educational settings.

## Materials and Methods

** Specimen collection and sampling period**

This research followed a descriptive, field-based study design. A non-probabilistic sampling method was used to collect a total of 80 water samples over four sampling rounds conducted across 6 months. Educational institutions were selected primarily based on their geographic location, with strategically geo-positioned schools in the municipalities of Cúcuta, Pamplona, and Los Patios included to ensure representative coverage of both urban and rural areas within the city of Cúcuta. Another selection criterion was that the sample should represent the different water supply sources found in these institutions since not all have the same infrastructure and storage capacity, which are critical variables in the project's outcomes. The participating institutions were organized sequentially to build a database containing the results obtained from each site. 

Samples were manually collected directly from the outlet valves of water dispensers and faucets after disinfection, using Whirl-Pak® bags, and transported to the laboratory under a cold chain at temperatures between 4 and 6 °C. Sample collection forms were duly completed. 

Another methodological approach used in the study to gather information on the condition of drinking water supply sources involved surveying personnel to learn about the condition of the storage tanks. Additionally, in situ variables such as temperature and pH were measured using pH testers (HANNA instruments). The process began by adding 10 ml of the water sample to a container. The device was then turned on and immersed in the sample for measurement. It is important to note that the equipment was calibrated before each use. 

A DPD checker disc (HANNA Instruments) was used to determine residual chlorine. Two cuvettes—one for the control and one for the sample—were each filled with 10 mL of water. A powder pillow of DPD free chlorine reagent (HI93701-0) was then added to the sample cuvette. Both cuvettes were capped and gently shaken for a few seconds before reading the result. 

**Microbiological analysis**

Detection of Escherichia coli and mesophilic aerobes. The membrane filtration method was used following the Colombian Technical Standard NTC 4772^21^, employing nitrocellulose membranes with a 0.45-µm pore size. A volume of 100 mL from each sample was filtered, and the membrane was then placed on Plate Count Agar (Merck, Frankfurt, Germany) for mesophilic aerobes growth and on Chromocult® Coliform Agar (Merck) for the detection of coliforms and Escherichia coli. Plates were incubated for 24 hours at 37 °C. 

Detection of Pseudomonas spp. In accordance with NTC 4940^22^, tubes with asparagine broth (Merck, Frankfurt, Germany) were inoculated and incubated at 37 °C for 48 hours. Tubes exhibiting turbidity and fluorescence were recorded as positive. For confirmation, 0.1 mL from each positive tube was streaked onto Cetrimide Agar (Oxoid, Hampshire, UK) and incubated at 37 °C for 24 hours. Oxidase tests were subsequently performed. 

Detection of Salmonella spp. The method outlined in the NTC 4574^23^ was used. A volume of 25 mL of the sample was added to 225 mL of peptone water (Acumedia, Madrid, Spain) and incubated at 37 °C for 24 hours. Subsequently, 0.1 mL of this pre-enrichment culture was transferred to Rappaport broth (Merck, Frankfurt, Germany) and incubated at 42 °C for 24 hours. Differential isolation was performed by streaking onto Hektoen Enteric Agar and SS Agar (Merck, Frankfurt, Germany), followed by incubation at 37 °C for 24 hours. The process concluded with biochemical confirmation. 

**Statistical analysis**

Descriptive measures were calculated for the physicochemical and microbiological variables, and the results were summarized using contingency tables to facilitate comparisons between municipalities. The data generated in this study were stored in the Mendeley Data repository, a platform developed by Elsevier for the storage of general data^24^. 

**Ethical considerations**

The research project was approved by the Vice-Rector's Office for Research at the Universidad de Santander, under initiation record No. 065-16 as part of the Internal Focused Call for Research and Technological Development Projects 2016-2017, identified by code PICF0216540231765EJ. Sample collection was authorized by the legal representatives of the educational institutions. 

## Results

According to the results of the physical and chemical tests of water condition regarding residual chlorine levels, no residual chlorine was found in 70.00% of the schools, indicating non-compliance with the established standards for this parameter. For the pH parameter, 87.50% of the water samples from the schools complied with the established ranges in 4 of the 4 sampling rounds, while 12.50% failed to meet the standards in 4 of the 4 sampling rounds. Surface water temperature averaged 18.8ºC in Pamplona, while in Cúcuta and Los Patios, the average was 26ºC, with variations depending on location, altitude, and atmospheric conditions (Table 1). 

**Hydrosanitary characteristics of the analyzed schools**

The information collected from school personnel and field observations made it possible to learn about water storage and treatment conditions within the educational institutions. From the data obtained, it was inferred that most schools are over 20 years old (95.00%). Only 40.00% of them reported information regarding improvements of water pipes, a common situation across the three municipalities. In 60.00% of the institutions, water tanks are installed above ground, mainly in the municipality of Pamplona, where all schools have this type of infrastructure. In contrast, underground tanks are more common in Los Patios (80.00%), while in the institutions located in Cúcuta, the proportion of overhead and underground tanks is similar. The predominant tank material is a combination of plastic and cement. This type of tank was observed more frequently in schools of Los Patios and Pamplona (80.00% and 60.00%, respectively). An important fact is that most of the water storage tanks in the institutions are covered with lids. Shortcomings were also identified in the frequency of maintenance, as most institutions reported performing it only once a year (55.00%), which poses a risk that is more frequent among schools located in Cúcuta. Regarding the cleaning and disinfection of the tanks, the most frequently used combination is chlorine and soap (35.00%). Most of the school personnel surveyed were able to recognize and identify the areas where water purification takes place. Only 20.00% of the schools reported conducting microbiological analysis of the water, and only 5.00% reported performing physical-chemical analyses (Table 2). 

**Table 1:** Physicochemical characteristics of drinking water from samples collected in situ

Physicochemical analysis in situ												
Code	Sampling 1			Sampling 2			Sampling 3			Sampling 4		
	T °C	pH	Chlorine	T °C	pH	Chlorine	T °C	pH	Chlorine	T °C	pH	Chlorine
1	17.3	7.3	0	16.7	7.3	0	21	8.6	0	21.3	9.5	0
2	16.5	7.4	1.5	17.8	7.2	0.9	17.2	8.8	0.2	18.2	8.6	1.5
3	18.6	7.1	0	19.1	7.3	0	16.9	9.3	0	18.9	8.8	0
4	16.5	6.5	0	17	6.8	0	16.2	9.7	0	17.6	9.4	0
5	16.8	7	1	17.5	7.5	1.2	17.4	8.4	0	19.2	8.4	0
6	26.9	7.2	0	29.5	8.5	0	27.2	8.6	0	25.9	8.6	0
7	10.1	8.8	0	28.3	9	0	10.7	9	0	26.6	8.7	0
8	27.2	8.8	0	28.1	8.9	0	25.7	8.4	0	25.8	9.2	0
9	27.5	8.1	2	29.1	8.2	2	25.4	7.8	2	28	8.4	2
10	27.9	8.6	0	29	8.5	0	27.5	8.5	0	26.5	8.8	0
11	26.6	7.8	0.9	27.1	9.1	0.7	25.3	8.7	1.5	27.2	7	2
12	26.3	8.2	1.5	27.1	8.6	0.5	25.7	8.6	1	26.9	7.3	1.2
13	27.2	7.1	1	26.9	8.6	1.5	26.5	8.5	1.2	27.3	8	1.2
14	26.8	8.9	0.1	27.7	9.1	0.2	26.4	8.5	0	27.8	8	0
15	25.2	5.7	0	11.9	8.5	0	27.1	8.6	0	21.9	8.2	0
16	23.6	8.5	0	24.6	9.2	0	17.1	8.8	0	21	8.9	0
17	29.9	8.8	0	28.4	9.2	0	27.1	8.7	0.5	27.1	8.6	0
18	26.5	7.7	0.3	27.1	8.6	1.2	26.1	8.5	1.7	26	8.3	0
19	21.5	8.6	0	22.9	8.4	0	25.7	8.4	0	23	8.3	0
20	27	9	0	23.4	8	0	26	9	0	25	8	0

Permissible limits (Resolution 2115 of 2007): Residual chlorine 0.3 to 2.0 mg/L pH between 6.5 to 9.0

**Table 2:** Water storage and treatment conditions of the schools studied

Variable	Municipality			
	Pamplona	Los Patios	Cúcuta	Total
Water piping improvements				
Yes (%)	40	40	40	40
No (%)	60	60	60	60
Water tank location				
Overhead (%)	100	20	60	60
Underground (%)	0	80	40	40
Storage tank materials				
Plastic (%)	40	20	30	30
Cement (%)	20	20	40	30
Plastic and cement (%)	60	80	30	50
Tanks are covered with lids				
Yes (%)	60	100	90	85
No (%)	40	0	100	60
Frequency of tank maintenance				
Every 4 months (%)	0	0	20	10
Every 6 months (%)	40	80	10	35
Every 12 months (%)	60	20	70	55
Products used for cleaning/disinfection				
Soap (%)	0	20	20	15
Chlorine (%)	20	40	40	35
Soap/chlorine (%)	40	40	30	35
Other (%)	40	0	10	15
Complaints from students				
Yes (%)	20	0	10	10
No (%)	80	100	90	90
Knowing where the water is made potable				
Yes (%)	60	100	90	85
No (%)	40	0	10	15
Knowing about laboratory analysis performed				
Yes (%)	40	0	20	20
No (%)	60	100	80	80
Performing temperature, pH, and residual chlorine analysis				
Yes (%)	0	0	10	5
No (%)	100	100	90	95

**Table 3:** Drinking water analysis for mesophilic aerobes, total coliforms, fecal coliforms, and Escherichia coli

School code	Aerobes maximum 100 CFU/100ml				Total coliforms 0 CFU /100 ml				Fecal coliforms 0 CFU /100 ml				E. coli 0 CFU /100 ml			
	Samplings				Samplings				Samplings				Samplings			
	1	2	3	4	1	2	3	4	1	2	3	4	1	2	3	4
1	10	0	44	50	0	0	32	25	0	0	1	0	0	0	0	0
2	49	116	152	120	0	0	0	0	0	0	0	0	0	0	0	0
3	16	100	16	20	0	0	0	0	0	0	0	0	0	0	0	0
4	12	160	112	105	0	24	20	18	0	12	1	0	0	0	0	0
5	*100	100	72	20	80	72	0	0	0	8	0	0	0	0	0	0
6	86	152	*100	*100	20	20	*100	8	20	12	0	0	0	0	0	0
7	*100	*100	0	*100	1200	84	0	240	0	3	0	40	0	0	0	0
8	*100	*100	*100	*100	488	336	0	48	0	0	0	4	0	0	0	0
9	37	51	*100	*100	0	0	*100	12	0	0	0	0	0	0	0	0
10	*100	*100	*100	*100	31	36	*100	80	0	2	16	0	0	0	0	0
11	*100	*100	*100	*100	30	1120	264	52	30	0	12	0	0	0	0	0
12	*100	992	*100	*100	2	280	0	0	1	0	0	0	0	0	0	0
13	*100	1920	*100	*100	12	112	16	0	4	0	0	0	0	0	0	0
14	98	*100	*100	*100	14	180	0	40	5	0	0	4	0	0	0	0
15	200	*100	*100	*100	90	100	2100	40	0	28	0	0	0	0	0	0
16	106	210	240	*100	4	10	8	*100	4	2	8	0	0	0	8	0
17	*100	18	392	*100	2	16	14	12	2	1	14	0	2	0	14	0
18	104	254	300	*100	29	0	12	8	29	10	12	0	29	0	12	0
19	86	301	1000	*100	86	5	40	35	0	5	40	35	0	0	40	0
20	*100	*100	*100	*100	*100	*100	*100	*100	20	140	182	150	0	0	0	0

* = Too numerous to count, and estimates were considered due to colony grouping

**Microbiological analysis**

Regarding the detection of microorganisms, this study identified atypical values across measurements, particularly in educational institutions located in Cúcuta. Based on the data, only 22.5% of the total samples analyzed met the permissible limit for microorganisms as established by current regulations (<100 colony-forming units [CFU]/100 mL)^25^. Only 25.00% of the schools complied with the established values at least once, and only one of the 20 institutions (a school located in Pamplona) met the standard in all four sampling rounds. In contrast, 45% of the schools failed to meet the standard in any of the four rounds (Table 3).

**Mesophilic aerobes**

 As shown in Table 3, the highest mesophilic aerobic counts—1920, 1000, and 992 CFU/100 mL—were recorded in water samples from schools 12, 13, and 19, all located in Cúcuta. These results indicate a higher level of contamination by mesophilic aerobes.

**Total and fecal coliforms**

Regarding the presence of coliforms, the situation is far from that observed with E. coli. Only two schools met the standard for total coliforms (i.e., absence in all four sampling rounds), representing just 10.00% of the schools analyzed. Considered differently, only 27.50% of the samples fell within the limits established by current regulations. 

 As expected, the findings regarding the presence of fecal coliforms in the drinking water of the schools studied were similar to those for total coliforms; in relation to the number of schools that showed no presence of these microorganisms in any of the 4 sampling rounds, only 15.00% of the institutions met this standard. However, from an overall perspective, 60.00% of the total samples complied with the regulations. Notably, all samples collected from school #20 exhibited particularly high CFU counts for these bacteria (Table 3).

**Escherichia coli**

In the cases where E. coli growth was observed, the samples always came from schools in the municipality of Cúcuta. The highest recorded value was 40 CFU/100 ml (Table 3). 

**Salmonella spp. and Pseudomonas spp**

Salmonella spp. is a microorganism strongly associated with the contamination of food or water sources. It was detected in only 10% of the schools studied, and only on a single occasion. Institution #20 showed the presence of Pseudomonas spp. in all samples analyzed (Table 4). 

Resolution 2115 of 2007^25^ does not mandate testing for Salmonella spp. and Pseudomonas spp. However, these analyses were conducted, and these microorganisms were detected in at least one school in each municipality during at least one of the four sampling rounds. Interestingly, mirroring the analysis performed to detect fecal coliforms, institution #20 showed the presence of Pseudomonas spp. in all samples (Table 4). The lowest average CFU counts for this bacterium were observed in schools located in the municipality of Pamplona. 

**Table 4:** Drinking water analysis for Salmonella spp. and Pseudomonas spp

School codes	Salmonella spp. Absence or presence in 25 ml				Pseudomonas aeruginos bact/ml (MPN)*			
	Sampling	Sampling	Sampling	Sampling	Sampling	Sampling	Sampling	Sampling
	1	2	3	4	1	2	3	4
1	A	A	A	A	0	0	9	9
2	A	A	A	A	0	0	43	43
3	A	A	A	A	0	7	43	43
4	A	A	A	A	0	0	0	0
5	A	A	A	A	0	0	0	0
6	A	A	A	A	0	0	0	0
7	A	A	A	A	0	0	21	21
8	A	A	A	A	0	0	23	0
9	A	A	A	A	0	0	23	0
10	A	A	A	A	0	0	11	0
11	A	A	A	A	0	0	21	0
12	A	A	A	A	0	0	21	0
13	A	A	A	A	0	0	93	0
14	P	A	A	A	0	0	0	21
15	P	A	A	A	7	7	0	0
16	A	A	A	A	0	2400	0	0
17	A	A	A	A	7	0	0	0
18	A	A	A	A	0	0	0	0
19	A	A	A	A	7	2400	0	0
20	A	A	A	A	7	7	7	7

* MPN: Most Probable Number. A: Absence. P: Presence.

## Discussion

The World Health Organization (WHO) Guidelines for Drinking Water Quality^26^, the European Union Council Directive 98/83/EC^27^, sanitary regulations from each country, and in Colombia, Resolution 2115 of 2007 issued by the Ministry of Health and Social Protection^25^, all establish that water intended for human consumption must meet specific physical, chemical, and microbiological standards. Water is fit for consumption if it is free from pathogenic microorganisms^28^. 

This study confirmed the presence of mesophiles at levels higher than those accepted, with a frequency of 77.5%. In other studies, mesophilic aerobes exceeded acceptable levels in 56% of the analyses performed by Pacheco Marza in 2023^29^. In comparison, Obando et al. affirmed that assessing water quality involves the evaluation of its chemical, physical, and biological characteristics in relation to its natural quality, anthropogenic impacts, and uses^30^. They also asserted that the detection of waterborne pathogens remains a global problem. Several countries in sub-Saharan Africa have adopted the WHO Guidelines for Drinking Water Quality (GDWQ) to guide the design of their sampling programs^31^. 

According to Omar et al. (2017), the World Health Organization (WHO) and the International Water Association (IWA) promote a preventive risk management approach to ensure the provision of safe drinking water through the implementation of “Water Safety Plans” (WSPs)^32^. These plans address all the stages of the water supply^32^. A study by Setty et al. in 2017 assessed the outcomes of WSP implementation in drinking water systems in France and Spain by analyzing quality and compliance indicators between 2003 and 2015^33^. Quality indicators included E. coli, fecal streptococci, total coliforms, heterotrophic plate counts, and disinfectants (residual and total chlorine). Implementing WSPs improved water quality and compliance with regulatory thresholds at most sites. Reported adverse effects were minimal, further supporting evidence that WSPs offer operational performance benefits^33^. Future research should focus on identifying the factors contributing to the successful implementation of WSPs and on determining best practices. Diduch et al.^34^ in 2016 noted that a group of microorganisms in groundwater are heterotrophic bacteria. Low concentrations of organic available matter in the environment (oligotrophic conditions) are conducive to these waterborne heterotrophic microorganisms. 

In Colombia, water quality requirements are imposed to ensure control and promote efforts to meet the conditions that make water safe for consumption. Suescún in 2021 mentioned that aerobic mesophilic bacteria present low counts, which reduces the likelihood of adverse effects on both consumers and water quality. This control on microorganisms is possible thanks to the concentration of chlorine^35^. 

In this study, coliforms and Escherichia coli were detected at levels above the limits established by the standard (0 CFU/100 mL) in at least one of the samples taken from every school, with the lowest average levels observed in Pamplona. Research conducted by Cueva in 2021, which aimed to remove total coliforms from stored water, found that the water consumed by residents of El Rosal de San Diego contained total coliforms and was unfit for human consumption according to the Maximum Permissible Limits (MPL) established in the Water Quality Regulation for Human Consumption (DS N°031-2010- SA36)^36^. The same findings applied to the presence of E. coli^37^. According to the study conducted by Estupiñan et al. 2020, which assessed bacteriological and physical characteristics of drinking water in the municipality of Une, Cundinamarca, Escherichia coli counts ranged between 32 and 65 CFU/100 ml in 10 samples (76.9%), indicating non-compliance with Colombian water quality regulations. The authors argue that the presence of fecal coliforms or E. coli in a water system is a sign of recent contamination by human or animal feces from sewers, septic systems, or animal enclosures^38^. Lucas et al., in Ecuador, reported that from the microbiological perspective, the presence of total and fecal coliforms posed a problem in all the localities studied, with values exceeding the permissible limits for water intended for human consumption^39^. Similarly, in a study conducted in Ecuador, observed contamination with Escherichia coli serotype O157:H7 in 80% of the water samples (40/50). 

There are environmental factors that cause water pollution, many of which are linked to human activity, such as discharges or waste from industrial processes, imperfect water treatment plants, leaking oil pipelines, petroleum derivatives, and improper disposal or dumping of waste, especially plastics^40^. This evidence confirms the need for a systematic and continuous review of the entire water supply network system, including the source, purification processes, distribution, adequate storage, along with control and monitoring. 

Among the enterobacteria capable of forming biofilms in drinking water distribution networks are the genera Shigella and Salmonella. These bacteria are responsible for diseases such as bacillary dysentery (in the case of Shigella), gastroenteritis (caused by Salmonella enterica serotype Typhimurium)^17^, and typhoid fever (caused by Salmonella enterica serotype Typhi). One of the objectives of this study was to detect Salmonella spp, given their important role in posing a threat to public health. Studies conducted in 2020 indicate that bacteria within biofilms live grouped in three-dimensional cellular clusters of aerobic and anaerobic bacterial cells. While many factors influence biofilm formation, the material used to construct pipes is one of the most important, as it affects not only the number of microorganisms but also the structure of the microbial community within the pipe. In addition, it is essential to understand each of these factors and their effect as a whole, in order to find solutions to biofilm-related problems in water distribution systems^41^,^42^. In 2017, Kovačić et al. successfully isolated Salmonella enterica subsp. enterica serovar Enteritidis from stool samples of patients who had consumed untreated water and groundwater^43^. Similarly, Douterlo et al. in 2018 inferred that biofilms offer several advantages to microorganisms, including the sharing of nutrients and metabolic byproducts, as well as enhanced resistance to environmental stressors^44^. Biofilms can directly impact the infrastructure of water distribution systems by promoting the biocorrosion of metal pipes. Additionally, they can alter the general characteristics of water, air, and soil. 

A high presence of Pseudomonas was observed in this study. This bacterium thrives at temperature between 30°C and 37°C, enabling it to survive in a wide range of environments and making it very easy to find in aquatic and terrestrial environments. These findings highlight the need for proper maintenance in water distribution systems^12^. The genus Pseudomonas is more resistant to chlorine than many other microorganisms and can inhibit coliforms, the most commonly used indicator of contamination. As a result, the probability of consuming water with a zero coliform count but inhibited by Pseudomonas increases, facilitating enteric colonization. A local source of contamination can become a regional health problem. Among the water samples analyzed, 68.75% had pH levels within the ranges established by Resolution 2115 of 2007^25^, and the temperature varied according to the municipality. Chlorine levels were found to be low in 50% of the institutions evaluated, and 10 of the 20 institutions failed to maintain the required residual chlorine level. This situation creates an ideal environment for bacterial growth and biofilm formation and correlates directly with the increased presence of mesophilic aerobes, total coliforms, fecal coliforms, and Pseudomonas aeruginosa. 

Although Colombian regulations do not require testing for Pseudomonas spp. and Salmonella spp. in drinking water, their inclusion in this study provides a more complete view of the microbiological quality of water in educational institutions. The aim is to set a precedent and encourage the replication of this approach in other broader scenarios. This comprehensive perspective is crucial for identifying multiple risks and designing effective mitigation strategies. 

## Conclusions

The study on the identification of pathogenic bacteria in drinking water in schools in Norte de Santander highlights the relevance of public health in protecting the most vulnerable populations. Implementing corrective measures based on these findings will not only support processes to improve water quality but will also enhance the health and well-being of students, thereby contributing to a safer and healthier school environment. 

Water storage conditions at each school were characterized using surveys and compliance profiles aligned with Colombian regulatory requirements. The physicochemical characteristics of the water were identified in situ by measuring residual chlorine levels, pH, and water temperature. Different data and instances of non-compliance of water quality were found that could lead to disease from the consumption of unsafe water.

Bacterial pathogens were identified using appropriate techniques, revealing failures in water reception and storage processes. The study also identified the need for greater monitoring of the filters installed for students to drink directly from these devices, as the findings of coliforms, Escherichia coli, Pseudomonas spp., and Salmonella spp., suggest possible cases of diseases not only among students but also among school staff. 

The findings of this study serve as input for implementing public policies and health programs aimed at improving water treatment infrastructure in schools. This includes training for maintenance personnel and the adoption of more advanced water treatment technologies. 

The identification of microbial contaminants underscores the importance of meeting water quality regulations. Shortcomings in the water purification process indicate the need to improve water treatment and storage practices. 
